# Optimal timing of ureteroscopic lithotripsy after the initial drainage treatment and risk factors for postoperative febrile urinary tract infection in patients with obstructive pyelonephritis: a retrospective study

**DOI:** 10.1186/s12894-020-00754-8

**Published:** 2021-01-15

**Authors:** Yoshitaka Itami, Makito Miyake, Takuya Owari, Takashi Iwamoto, Daisuke Gotoh, Hitoshi Momose, Kiyohide Fujimoto, Shuya Hirao

**Affiliations:** 1 Department of Urology, Hirao Hospital, 6-28 Hyobu-cho, Kashihara, Nara 634-0076 Japan; 2 Department of Urology, Nara Medical University, 840 Shijo-cho, Kashihara, Nara 634-8522 Japan

**Keywords:** Ureteroscopic lithotripsy, Febrile urinary tract infection, Obstructive pyelonephritis, Preoperative ureteral stent placement period, Operation time

## Abstract

**Background:**

A history of preoperative obstructive pyelonephritis has been reported as a risk factor for febrile urinary tract infection (fUTI) after ureteroscopic lithotripsy (URSL). But there is no clear evidence of risk factors for developing fUTI including the optimal timing of URSL after obstructive pyelonephritis treatment.

**Methods:**

Of the 1361 patients, who underwent URSL at our hospital from January 2011 to December 2017, 239 patients had a history of pre-URSL obstructive pyelonephritis. The risk factors were analyzed by comparing the patients’ backgrounds with the presence or absence of fUTI after URSL. The factors examined were age, gender, body mass index, comorbidity, presence or absence of preoperative ureteral stent, stone position, stone laterality, stone size, Hounsfield unit (HU) value on computed tomography scan, history of sepsis during obstructive pyelonephritis, period from antipyresis to URSL, ureteral stenting period, operation time, and presence or absence of access sheath at URSL. In addition, the stone components and renal pelvic urinary culture bacterial species during pre-URSL pyelonephritis were also examined.

**Results:**

Post-URSL fUTI developed in 32 of 239 patients (13.4%), and 11 of these 32 cases led to sepsis (34.4%). Univariate analysis showed that stone position, stone maximum HU value, presence of sepsis during obstructive pyelonephritis, period from antipyresis to URSL, pre-URSL ureteral stent placement, operation time were risk factors of fUTI. Stone components and urinary cultures during pyelonephritis were not associated with risk of fUTI. Multivariate analysis showed that renal stone position, pre-URSL ureteral stent placement > 21 days, and operation time > 75 min were independent risk factors of fUTI following the URSL.

**Conclusions:**

F-UTI following the URSL could be avoided by ureteral stent placement period 21 days or less and operation time 75 min or less in patients with obstructive pyelonephritis.

## Background

Obstructive pyelonephritis related to ureteral stones can be a fatal urological emergency [1, 2]. Obstructive pyelonephritis is often severe and can lead to severe sepsis and disseminated intravascular coagulation [3]. The mortality rate of patients with sepsis due to acute complicated pyelonephritis has been reported to be about 2% [4]. Emergency urinary drainage using a ureteral stent or nephrostomy and subsequent stone treatment is crucial in treating these patients [1, 2]. However, a history of preoperative obstructive pyelonephritis and ureteral stent placement has been reported as a risk factor for febrile urinary tract infection (fUTI) after ureteroscopic lithotripsy (URSL) [5, 6].

It has not been established whether URSL can be safely performed after renal drainage; there are concerns that URSL may cause post-URSL fever or sepsis due to increased renal pressure. Furthermore, the optimal timing of URSL after drainage and risk factors for post-URSL fUTI has not been established. The elucidation of patient-, stone-, and operative-factors associated with increased risk of post-URSL fUTI is important to be able to identify high-risk patients, advise on individual risks before operation, and reduce the incidence of fUTI. The purpose of this study is to establish the optimal timing of URSL and risk factors for fUTI following URSL in patients with obstructive pyelonephritis.

## Methods

### Patient data collection

The methods and procedures for this study were approved by the Ethics Committee of Hirao Hospital (project identification code: 2019–4). The retrospective analyses evaluated data from 1361 patients who underwent URSL at Hirao Hospital from January 2011 to December 2017. There was no history of pre-URSL pyelonephritis, ureteral stone of ileal conduit, or transplant renal stone in 1122 of these patients, leaving 239 patients with a history of pre-URSL obstructive pyelonephritis eligible for this study. All patients underwent urinary drainage by insertion of a ureteral stent or nephrostomy.

The diagnosis of obstructive pyelonephritis was based on computed tomography (CT) and laboratory data, such as bacteriuria and leukocyturia. According to criteria established by the American College of Chest Physicians/Society of Critical Care Medicine Consensus Conference Committee [7], systemic inflammatory response syndrome (SIRS) was defined by the presence of ≥ 2 of the following features: (1) body temperature lower than 36 °C or higher than 38 °C (2) heart rate > 90 beats per minute; (3) respiratory rate > 20 breaths per minute or PaCO_2_ less than 32 mmHg; (4) a white blood cell count higher than 12,000 per mm^3^ or lower than 4,000 per mm^3^. In this study, fUTI accompanied with SIRS were defined as sepsis based on an international definition [8].

### Treatment policy of obstructive pyelonephritis

The first line treatment for obstructive pyelonephritis is urinary drainage, which is mainly performed by ureteral stenting. Retrograde placement of a 6 Fr ureteral stent was performed using a flexible cystoscope under transurethral local anesthesia. In case with highly viscosity of the renal pelvic urine, we used a single J stent that can clean the renal pelvis because of a high risk of ureteral stent occlusion. If retrograde placement was not possible, nephrostomy using an 8.3 Fr pigtail stent (Jinro, Boston Scientific, Tokyo, Japan) was performed under ultrasound and fluoroscopy. In most cases, renal pelvic urine cultures were submitted, and empirical antibiotic treatment was administered for at least 14 days. The period between pyelonephritis treatment and URSL was based on the judgment of each physician. The day of antipyresis was defined as the first day of body temperature less than 37 °C, which continued for two or more days.

### URSL procedure

All URSLs were performed under general anesthesia in the lithotomy position. Ureteral stent was removed at the beginning of URSL. A semi-rigid 6.4/7.8 Fr ureteroscope (Olympus, Tokyo, Japan) was inserted into the ureter along with a flexible 0.035 inch guidewire. If there were only distal ureteral stones, they were broken up with a holmium: yttrium–aluminum-garnet laser (VersaPulse; Lumenis, Tel Aviv, Israel) using a semi-rigid ureteroscope and collected with basket forceps. If there were renal stones, a ureteral access sheath (UAS) (Flexor, 12/14 Fr, COOK, Tokyo, Japan; Bi-Flex, 12/14 Fr or 10/12 Fr, ROCAMED, Mclean, VA, USA) was inserted into the proximal ureter along with the guidewire under fluoroscopy. A part of upper ureteral stones were pushed back into the renal pelvis during URSL, and a UAS was inserted to perform intrarenal surgery. The flexible ureteroscope (URF-P5, Olympus) was then inserted through the UAS, and stones were crushed by the same methods of semi-rigid ureteroscopy. Ureteral stents and urethral catheters were placed after all ureteroscopic procedures. If there was no fever after URSL, urethral catheters were removed the day after operation, and ureteral stents were removed 7–14 days after operation outpatiently. Pre-URSL antibiotic prophylaxis was based on the judgment of each physician. Mostly perioperative antibiotics were infused with second or third generation cephems for 3 days from the operation date. Based on the results of preoperative renal pelvic urine culture, the antibiotic was appropriately changed to a sensitive antibiotic. Post-URSL fUTI was defined as a fever of 38 °C or higher accompanied by pyuria or bacteriuria within 7 days of surgery.

### Analysis of potential risk factors

The parameters investigated as potential risk factors included age, sex, body mass index (BMI), comorbidity (diabetes, hypertension, hyperlipidemia), presence or absence of pre-URSL ureteral stent, stone position at URSL, stone laterality, stone size, Hounsfield unit (HU) value, history of sepsis during obstructive pyelonephritis, renal pelvic urine culture at urinary drainage, period from antipyresis to URSL, pre-URSL ureteral stent placement period, operation time, and presence or absence of UAS at URSL. In addition, the stone components and renal pelvic urinary culture bacterial species at urinary drainage during pre-URSL pyelonephritis were also examined.

### Statistical analysis

Data are shown as bar charts or dot plots and were evaluated using the Student t-test, the Mann–Whitney U test, or the χ^2^ test, as appropriate. Multivariate analyses were used to identify independent prognostic variables based on logistic analysis (p < 0.05 in the univariate analyses). Multivariate analyses were performed using StatMate (version 5.0; Tokyo, Japan) and other data were analyzed using PRISM software (version 7.00; San Diego, CA, USA). A p value < 0.05 was considered statistically significant.

## Results

Of the 239 patients with pre-URSL obstructive pyelonephritis, 222 (92.9%) had a ureteral stent, of which 205 were double J stents and 17 were single J stents, and 17 (7.1%) had a nephrostomy during urinary drainage. Pre-URSL pyelonephritis with concurrent sepsis was detected in 86 of 239 patients (36.0%). Post-URSL fUTI developed in 32 of 239 patients (13.4%). Post-URSL fUTI led to sepsis in 11 of these 32 patients (34.4%). Table 1 shows the perioperative characteristics of the 239 patients and a comparison between those with post-URSL fUTI and those without. 8 patients had stones with a diameter of 2 cm or more and recommended PNL, but URSL was performed due to patient preferences such as limited hospital stay for work. There were no significant differences in age, sex, BMI, comorbidities, or stone size between the two groups. 149 patients used UAS during URSL, 140 patients (94.0%) were 12/14 Fr, 9 patients (6.0%) were 10/12 Fr, and there was no significant difference in post-URSL fUTI incidence with UAS size. The fUTI group presented with renal stones at URSL, had a higher maximal HU value, higher incidence of concurrent sepsis during obstructive pyelonephritis, longer period from antipyresis to URSL, longer period of pre-URSL ureteral stent placement, and longer operation time than the non-fUTI group. However, the initial stone-free rate was similar between the two groups (90.8% versus 90.6%). There was no significant difference in the number of patients administered pre-URSL prophylactic antibiotics between the two groups. Furthermore, there was no significant difference in fUTI between nephrostomy or ureteral stent, and also between single J stent or double J stent. Table 2 compares the perioperative characteristics of 239 patients, divided into ureteral and renal stones. 164 patients (68.6%) had ureteral stones and 75 patients (31.4%) had renal stones. UAS was used in all 75 cases of renal stones. In 74 of 99 cases of upper ureteral stones, the stones were pushed back into the renal pelvis from the ureter and were performed intrarenal surgery. Patients with renal stones were significantly older, and had more DM and HT comorbidities, higher stone sizes and HU values than those with ureteral stones. Moreover, operation time of URSL was significantly longer for renal stones than for ureteral stones. The initial stone-free rate of renal stones was significantly lower than ureteral stones (78.7% versus 96.3%).

**Table 1:** Patients characteristics of URSL with or without post-URSL fUTI

Variables	fUTI − (N = 207)	fUTI + (N = 32)	p value
Age			
Median (range), years	66 (19–89)	66 (35–90)	0.97
Gender			
Male/female (%)	110/97 (53.1)	13/19 (40.6)	0.19
Body mass index			
Median (range), kg/m^2^	23.5 (14.3–41.8)	23.9 (15.9–37.9)	0.74
Comorbidities			
Diabetes yes/no (%)	49/158 (23.7)	5/27 (15.6)	0.31
Hypertension yes/no (%)	88/127 (42.5)	15/17 (46.9)	0.64
Hyperlipidemia yes/no (%)	30/177 (14.5)	6/26 (18.8)	0.53
Pre-URSL sepsis			
Yes/no (%)	69/138 (33.3)	17/15 (53.1)	0.01
Stone position at URSL			
Lower/mid/upper ureter/renal (%)	48/14/89/56 (23.2)/(6.8)/(43.0)/(27.0)	0/3/10/19 (0)/(9.3)/(31.2)/(59.5)	0.002
Stone laterality			
Right/left/bilateral (%)	90/113/4 (43.5)/(54.6)/(1.9)	14/16 /2 (43.7)/(50.0)/(6.3)	0.54
Stone size (maximal diameter)			
Median (range), mm	7.7 (2.5–49.2)	9.5 (5.5–44.6)	0.98
Maximal hounsfield unit value			
Median (range), HU	651 (163–1999)	875 (320–1593)	0.034
Pre-URSL renal pelvic urine culture			
Positive/negative/not evaluated (%)	83/104/20 (40.1)/(50.2)/(9.7)	13/18/1 (40.6)/(56.3)/(3.1)	0.4
Pre-URSL blood culture			
Positive/negative/not evaluated (%)	23/63/121 (11.1)/(30.4)/(58.5)	4/13/15 (12.5)/(40.6)/(46.9)	0.45
Pre-URSL prophylactic antibiotics			
Yes/no (%)	39/168 (18.8)	8/24 (33.3)	0.41
Operation time			
Median (range), min	57.0 (14–220)	81.5 (21–147)	< 0.001
Pre-URSL ureteral stenting			
Yes/no (%)	192/15 (92.8)	30/2 (93.8)	0.41
Pre-URSL nephrostomy			
Yes/no (%)	15/192 (7.2)	2/30 (6.2)	0.66
Period from antipyresis to URSL			
Median (range), days	13.5 (1–83)	21.0 (2–90)	0.043
Pre-URSL ureteral stenting placement period			
Median (range), days	20 (0–124)	28.5 (2–117)	0.003
Using access sheath during URSL			
Yes/no (%)	124/83 (59.9)	25/7 (78.1)	0.047
Initial stone free rate			
Yes/no (%)	188/19 (90.8)	29/3 (90.6)	0.97

*fUTI* febrile urinary tract infection, *CT* computed tomography, *URSL* ureteroscopic lithotripsy

**Table 2:** Patients characteristics of URSL according to stone position, ureteral or renal stones

Variables	Ureteral stone (N = 164)	Renal stone (N = 75)	p value
Age			
Median (range), years	65 (26–90)	69 (19–89)	0.011
Gender			
Male/female (%)	88/76 (53.7)	35/40 (46.7)	0.32
Body mass index			
Median (range), kg/m^2^	23.5 (14.3–37.2)	23.6 (15.9–41.8)	0.4
Comorbidities			
Diabetes yes/no (%)	30/134 (18.3)	24/51 (32.0)	0.019
Hypertension yes/no (%)	61/103 (37.2)	42/33 (56.0)	0.006
Hyperlipidemia yes/no (%)	22/142 (13.4)	14/61 (18.7)	0.29
Pre-URSL sepsis			
Yes/no (%)	51/113 (31.1)	33/42 (44.0)	0.05
Stone laterality			
Right/left/bilateral (%)	64/94/6 (39.0)/(57.3)/(3.7)	40/35 /0 (53.3)/(46.6)/(0)	0.044
Stone size (maximal diameter)			
Median (range), mm	7.2 (2.5–36.3)	11.2 (3.1–49.2)	< 0.0001
Maximal hounsfield unit value			
Median (range), HU	618 (163–1593)	843 (293–1999)	< 0.0001
Pre-URSL renal pelvic urine culture			
Positive/negative/not evaluated (%)	64/87/13 (39.0)/(53.0)/(8.0)	32/35/8 (42.7)/(46.7)/(10.7)	0.6
Pre-URSL blood culture			
Positive/negative/not evaluated (%)	14/56/94 (8.5)/(34.1)/(57.3)	13/20/42 (17.3)/(27.1)/(56.0)	0.11
Pre-URSL prophylactic antibiotics			
Yes/no (%)	30/134 (18.3)	17/58 (22.7)	0.43
Operation time			
Median (range), min	50.0 (14–220)	72.0 (25–147)	< 0.0001
Pre-URSL ureteral stenting			
Yes/No (%)	149/15 (91.5)	73/2 (97.3)	0.07
Pre-URSL nephrostomy			
Yes/no (%)	15/149 (8.5)	2/73 (2.7)	0.07
Period from antipyresis to URSL			
Median (range), days	13.0 (1–79)	17.0 (3–90)	0.018
Pre-URSL ureteral stenting placement period			
Median (range), days	20 (0–124)	24 (2–117)	0.11
Using access sheath during URSL			
Yes/no (%)	74/90 (45.1)	75/0 (100)	< 0.0001
Post-URSL futi			
Yes/no (%)	13/151 (7.9)	19/56 (25.3)	< 0.0001
Initial stone free rate			
Yes/no (%)	158/6 (96.3)	59/16 (78.7)	< 0.0001

*fUTI* febrile urinary tract infection, *CT* computed tomography, *URSL* ureteroscopic lithotripsy

Multivariate analysis in all patients showed that renal stone position at URSL, pre-URSL ureteral stent placement period > 21 days, and operation time > 75 min were independent risk factors (Table 3). In Table 2, it was shown that the operation time was significantly longer in renal stone patients than in ureteral stone patients, so the risk factors were examined only in renal stone patients. Similar to Table 3, pre-URSL ureteral stent placement period > 21 days, and operation time > 75 min were independent risk factors for renal stone patients only (Table 4).

**Table 3:** Risk factors for fUTI following URSL performed after obstructive pyelonephritis treatment

Variables	Univariable analysis^a^			Multivariable analysis^a^		
	Odds ratio	95% CI	p value	Odds ratio	95% CI	p value
Gender						
Male	1					
Female	1.66	0.78–3.53	0.19			
Pre-URSL sepsis						
No	1			1		
Yes	2.75	1.29–5.86	0.009	2.08	0.89–4.83	0.089
Stone position						
Ureter	1					
Renal	3.94	1.83–8.51	< 0.001	2.91	1.29–6.58	0.01
Maximal Hounsfield unit value (HU)^b^						
< 750	1			1		
≥ 750	2.79	1.13–6.87	0.026	1.15	0.49–2.68	0.74
Period from antipyresis to URSL (days)^b^						
< 20	1					
≥ 20	2.23	1.02–4.86	0.043			
Pre-URSL ureteral stent placement period (days)^b^						
< 21	1			1		
≥ 21	4.22	1.65–10.8	0.003	2.77	1.12–6.8	0.028
Operation time (min)^b^						
< 75	1			1		
≥ 75	6.75	3.05–14.9	< 0.001	3.37	1.45–7.84	0.005
Using access sheath during URSL						
No	1					
Yes	2.39	0.99–5.78	0.053			

*fUTI* febrile urinary tract infection, *CT* computed tomography, *HU* hounsfield unit, *URSL* ureteroscopic lithotripsy

^a^Logistic analysis

^b^Cutt off value of continuous variable is calculated from ROC curve

**Table 4:** Risk factors for fUTI in renal stones following URSL performed after obstructive pyelonephritis treatment

Variables	Univariable analysis^a^			Multivariable analysis^a^		
	Odds ratio	95% CI	p value	Odds ratio	95% CI	p value
Gender						
Male	1					
Female	1.99	0.69–5.75	0.20			
Pre-URSL sepsis						
No	1					
Yes	2.13	0.74–6.11	0.24			
Maximal Hounsfield unit value (HU)^b^						
< 750	1					
≥ 750	1.12	0.39–3.17	0.84			
Period from antipyresis to URSL (days)^b^						
< 20	1					
≥ 20	0.9	0.31–2.64	0.85			
Pre-URSL ureteral stent placement period (days)^b^						
< 21	1			1		
≥ 21	4.3	1.13–16.4	0.033	5.16	1.28–20.8	0.021
Operation time (min)^b^						
< 75	1			1		
≥ 75	3.35	1.11–10.1	0.032	4.00	1.25–12.8	0.020

*curvefUTI* febrile urinary tract infection, *CT* computed tomography, *HU* hounsfield unit, *URSL* ureteroscopic lithotripsy

^a^Logistic analysis

bCutt off value of continuous variable is calculated from ROC

The bacterial species found in renal pelvic urinary cultures at urinary drainage during obstructive pyelonephritis and the stone components were not associated with post-URSL fUTI (Tables 5 and 6).

**Table 5:** Renal pelvic urinary culture bacterial species at drainage during pre-URSL pyelonephritis with or without post-URSL fUTI

	fUTI −		fUTI +	
	n	%	n	%
Negative	104	50.2	18	56.3
Escherichia coli	35	17.2	4	12.5
Enterococcus faecalis	18	8.7	1	3.1
Pseudomonas aeruginosa	9	4.3	2	6.3
Staphylococcus aureus	7	3.4	0	0
Klebsiella pneumoniae	6	2.9	1	3.1
Proteus mirabilis	5	2.4	2	6.3
Streptococcus agalactiae	4	1.9	0	0
Morganella morganii	1	0.5	0	0
Others	18	8.7	4	12.5
Total	207		32	

*URSL* ureteroscopic lithotripsy, *fUTI* febrile urinary tract infection

**Table 6:** Stone component with or without post-URSL fUTI

	fUTI −		fUTI +	
	N	%	n	%
Ca oxalate	136	65.7	22	68.8
Ca phosphate	10	4.8	3	9.4
NH4MgPO4	24	11.6	2	6.3
Carbonate apatite	2	1	2	6.3
Uric acid	7	3.4	0	0
Not evaluated	28	13.5	3	9.4
Total	207		32	

*URSL* ureteroscopic lithotripsy, *fUTI* febrile urinary tract infection

**Fig. 1:**
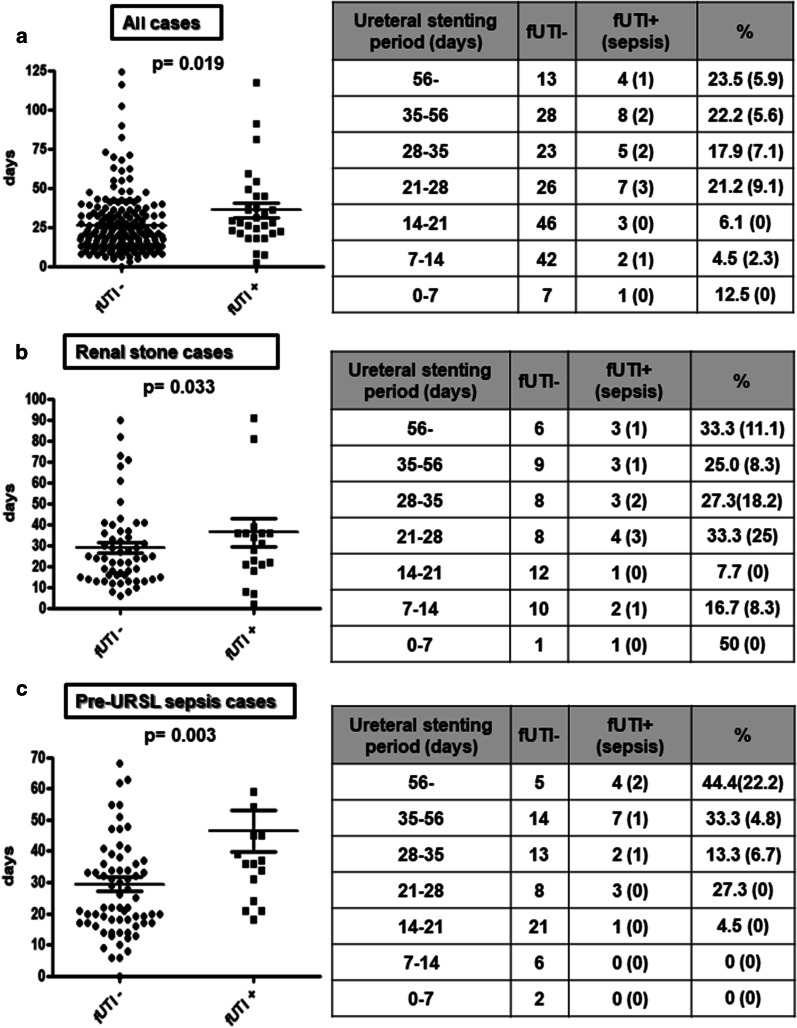
Post-URSL fUTI incidence depending on pre-URSL ureteral stent placement period. **a** Comparison of pre-URSL ureteral stent placement periods with or without post-URSL fUTI and post-URSL fUTI incidence according to pre-URSL ureteral stent placement in all cases except seven cases when insertion was unknown. **b** Same examination as (**a**) in renal stone cases. **c** Same examination as (**a**) in pre-URSL with sepsis cases. *fUTI* febrile urinary tract infection, *URSL* ureteroscopic lithotripsy

The pre-URSL ureteral stent placement period both in all cases and renal stone cases was significantly longer in the post-URSL fUTI group (Fig. 1a, b). Furthermore, the incidence of post-URSL fUTI significantly increased when the stent placement period was over 21 days. Similarly, in cases with pre-URSL concurrent with sepsis, the incidence of fUTI increased significantly after 21 days, and increased further after 35 days. No post-URSL fUTI with sepsis was found within 28 days of stent placement (Fig. 1c).

**Fig. 2:**
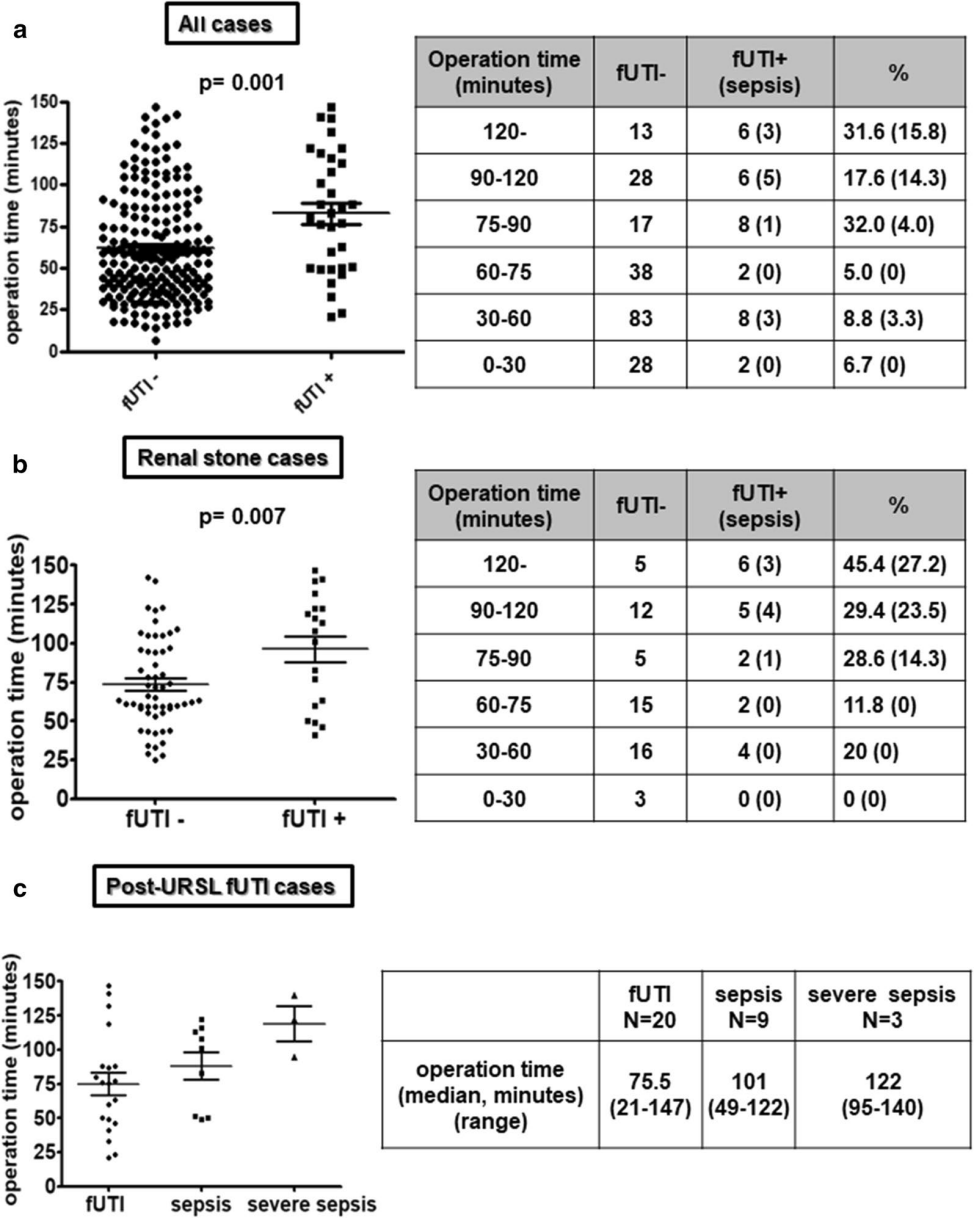
Post-URSL fUTI incidence depending on operation time. **a** Comparison of operation time with or without post-URSL fUTI and post-URSL fUTI incidence according to operative time. **b** Same examination as (**a**) in renal stone cases. **c** Comparison of operation time by severity in post-URSL fUTI cases. Severe sepsis was defined as concurrent disseminated intravascular coagulation (DIC) or as a condition requiring a vasopressor. *fUTI* febrile urinary tract infection, *URSL* ureteroscopic lithotripsy

With respect to the duration of the operation both in all cases and renal stone cases, the frequency of post-URSL fUTI increased significantly after an operation > 75 min, and post-URSL fUTI with sepsis increased following an operation > 90 min (Fig. 2a, b). The length of the operation showed a correlation with the severity of post-URSL fUTI (Fig. 2c).

**Fig. 3:**
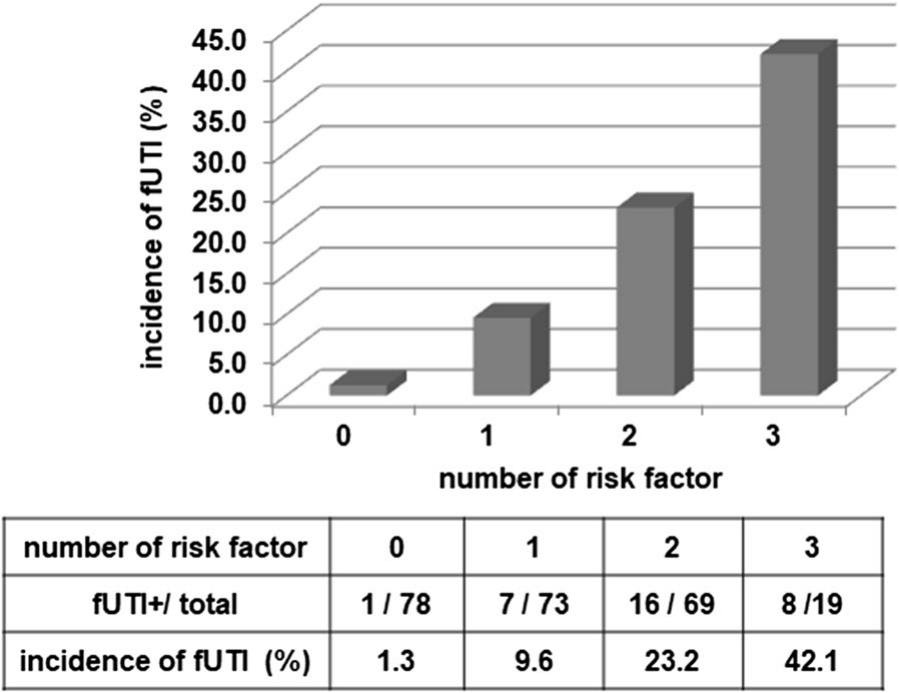
Post-URSL fUTI incidence stratified by three risk factors, renal stone position at URSL, pre-URSL ureteral stent placement period > 21 days, and operation time > 75 min. The number of risk factors were defined as the point. *URSL* ureteroscopic lithotripsy, *fUTI* febrile urinary tract infection

Three risk factors were used to stratify patient groups: renal stone position at URSL, pre-URSL ureteral stent placement period > 21 days, and operation time > 75 min. Patients with a higher number of risk factors showed a significantly increased incidence of post-URSL fUTI (Fig. 3).

## Discussion

Risk factors for fUTI after URSL have been reported as the presence of pre-URSL pyuria, a history of pre-URSL pyelonephritis, pre-URSL ureteral stent placement, and operation duration of 90 min or more [5, 9–11]. Youssef et al. reported that URSL in patients with a history of pre-URSL pyelonephritis with sepsis resulted in an increase in complications, including post-URSL fUTI, prolonged hospital stays, and prolonged post-URSL antibiotic administration [6]. A ureteral stent is often used for urinary drainage in stone-associated pyelonephritis, but there are no studies examining the relationship between the ureteral stenting period and post-URSL fUTI.

In this study, a risk factor for the development of fUTI following treatment of obstructive pyelonephritis and URSL was identified as > 21 days of ureteral stenting (Fig. 1a). Pre-stenting can improve the passive dilation of the ureter and increase the stone-free rate during URSL [12, 13]. It has been reported that bacteriuria and bacterial colonization of the stent occurs over time after stent placement [14]. Bacterial colonization of the stent was not observed within 2 weeks but was observed in 23.5–33% of patients after less than 4 weeks, 33.3–50% after 4–6 weeks, and 71.4% after more than 6 weeks [15]. Therefore, the optimal timing of URSL appears to be 7–21 days following stent placement.

An increased duration of operation was correlated with higher the risk of fUTI. Some studies have indicated that the risk of fUTI increases after an operation > 90 min [5, 11], but in this study the risk increased after operations of 75 min (Fig. 2). The reason for this may be that URSL performed after pyelonephritis poses a higher risk of bacterial exposure, so shorter operation times would reduce the incidence of fUTI. Furthermore, this study also showed that post-URSL sepsis complications increased with an operation time of > 90 min. These results indicate that the optimal duration of URSL after pyelonephritis should be 75 min or less, and operations longer than 90 min should be avoided in order to prevent severe fUTI.

Regarding the stone position, renal stones were considered to increase the intrapelvic pressure during URSL and cause fUTI regardless of the use of UAS [16]. However, in this study, there was no significant difference in the incidence of fUTI depending on the diameters of the access sheath. The reason for the high fUTI in patients with renal stones is considered to be that they are older than patients with ureteral stones, have many comorbidities such as DM, and have a large stone size and a long operation time, as shown in Table 2.

It has been previously reported that administration of prophylactic antibiotics reduced the rate of post-URSL bacteriuria, but the post-URSL fUTI incidence rate was not significantly different to the control group [17]. In a recent systematic review, it was reported that prophylactic antibiotics showed a certain effect [18]. In this study, no effect of prophylactic antibiotics on fUTI onset was observed, with similar results seen in selective cases of pre-URSL pyelonephritis with sepsis. However, the criteria for prophylactic antibiotic administration were not constant in this study, It is considered that prophylactic antibiotics should be more aggressively administered in the future to prevent the fUTI development, especially in patients with ureteral stent for > 21 days and renal stones with large stones. The Infectious Diseases Society of America guidelines state that replacing long-term indwelling catheters immediately before surgery is more effective than administering antibiotics [19]. Therefore, when URSL is performed on a patient with a ureteral stent that has been indwelling for > 21 days, it is recommended that the stent be replaced before URSL. However, the replacement procedure itself is mildly invasive, and there is a slight risk of fUTI, so indication needs to be considered.

This study has limitations, which must be considered. First, our retrospective analysis of data is from a single institution and of a small sample size, which are associated with a high risk of selection bias. Second, the types and duration of antibiotics administered during pyelonephritis and the prophylactic antibiotics before URSL were dependent on each attending physician and were not standardized. However, these factors would not increase the risk of post-URSL fUTI and were considered to have little effect on the results of the study.

## Conclusions

It is suggested that fUTI following URSL could be avoided by compliance with a ureteral stent placement period of 21 days or less and operation time of 75 min or less in patients with obstructive pyelonephritis, especially in renal stone cases.

## Data Availability

The data that support the findings of this study are available on request from the corresponding author Y.I. The data are not publicly available due to them containing information that could compromise research participant privacy.

## Abbreviations

BMI: Body mass index
CT: Computed tomography
fUTI: Febrile urinary tract infection
HU: Hounsfield unit
SIRS: Systemic inflammatory response syndrome
UAS: Ureteral access sheath
URSL: Ureteroscopic lithotripsy
